# Post–COVID-19 Conditions Among Children 90 Days After SARS-CoV-2 Infection

**DOI:** 10.1001/jamanetworkopen.2022.23253

**Published:** 2022-07-22

**Authors:** Anna L. Funk, Nathan Kuppermann, Todd A. Florin, Daniel J. Tancredi, Jianling Xie, Kelly Kim, Yaron Finkelstein, Mark I. Neuman, Marina I. Salvadori, Adriana Yock-Corrales, Kristen A. Breslin, Lilliam Ambroggio, Pradip P. Chaudhari, Kelly R. Bergmann, Michael A. Gardiner, Jasmine R. Nebhrajani, Carmen Campos, Fahd A. Ahmad, Laura F. Sartori, Nidhya Navanandan, Nirupama Kannikeswaran, Kerry Caperell, Claudia R. Morris, Santiago Mintegi, Iker Gangoiti, Vikram J. Sabhaney, Amy C. Plint, Terry P. Klassen, Usha R. Avva, Nipam P. Shah, Andrew C. Dixon, Maren M. Lunoe, Sarah M. Becker, Alexander J. Rogers, Viviana Pavlicich, Stuart R. Dalziel, Daniel C. Payne, Richard Malley, Meredith L. Borland, Andrea K. Morrison, Maala Bhatt, Pedro B. Rino, Isabel Beneyto Ferre, Michelle Eckerle, April J. Kam, Shu-Ling Chong, Laura Palumbo, Maria Y. Kwok, Jonathan C. Cherry, Naveen Poonai, Muhammad Wassem, Norma-Jean Simon, Stephen B. Freedman

**Affiliations:** 1 Department of Pediatrics, Cumming School of Medicine, University of Calgary, Calgary, Alberta, Canada; 2 Department of Emergency Medicine, University of California, Davis School of Medicine, Sacramento; 3 Department of Pediatrics, University of California, Davis School of Medicine, Sacramento; 4 Department of Pediatrics, Feinberg School of Medicine, Northwestern University, Chicago, Illinois; 5 Division of Emergency Medicine, Ann and Robert H. Lurie Children’s Hospital of Chicago, Chicago, Illinois; 6 Division of Emergency Medicine, Department of Pediatrics, Hospital for Sick Children, Toronto, Ontario, Canada; 7 Division of Clinical Pharmacology and Toxicology, Department of Pediatrics, Hospital for Sick Children, Toronto, Ontario, Canada; 8 Department of Pediatrics, Harvard Medical School, Boston, Massachusetts; 9 Division of Emergency Medicine, Boston Children’s Hospital, Boston, Massachusetts; 10 Department of Pediatrics, McGill University, Montreal, Quebec, Canada; 11 Emergency Department, Hospital Nacional de Niños “Dr. Carlos Sáenz Herrera,” CCSS, San José, Costa Rica; 12 Department of Emergency Medicine and Trauma Services, Children's National Hospital, Washington, DC; 13 Department of Pediatrics, University of Colorado, Aurora; 14 Section of Emergency Medicine, Children’s Hospital Colorado, Aurora; 15 Division of Emergency and Transport Medicine, Children's Hospital Los Angeles and Keck School of Medicine of the University of Southern California, Los Angeles; 16 Department of Emergency Medicine, Children’s Minnesota, Minneapolis; 17 Department of Pediatrics, University of California, San Diego, Rady Children’s Hospital, San Diego; 18 Department of Pediatrics, St Mary’s Medical Center, West Palm Beach, Florida; 19 Pediatric Emergency Department, Hospital Universitario Miguel Servet, Zaragoza, Spain; 20 Department of Pediatrics, Washington University in St Louis School of Medicine, St Louis, Missouri; 21 Division of Pediatric Emergency Medicine, Department of Pediatrics, Children’s Hospital of Philadelphia, Philadelphia, Pennsylvania; 22 Division of Emergency Medicine, Children’s Hospital of Michigan, Detroit; 23 Department of Pediatrics, Central Michigan University, Mt Pleasant; 24 Department of Pediatrics, University of Louisville, Louisville, Kentucky; 25 Department of Pediatrics, Norton Children’s Hospital, Louisville, Kentucky; 26 Department of Pediatrics, Division of Pediatric Emergency Medicine, Emory University School of Medicine, Children’s Healthcare of Atlanta, Atlanta, Georgia; 27 Pediatric Emergency Department, Biocruces Bizkaia Health Research Institute, Hospital Universitario Cruces, University of the Basque Country, UPV/EHU Bilbao, Basque Country, Spain; 28 Department of Paediatrics, University of British Columbia, Vancouver, British Columbia, Canada; 29 Division of Emergency Medicine, Children’s Hospital of Eastern Ontario, Ottawa, Ontario, Canada; 30 Department of Pediatrics, University of Ottawa, Ottawa, Ontario, Canada; 31 Department of Emergency Medicine, University of Ottawa, Ottawa, Ontario, Canada; 32 Children’s Hospital Research Institute of Manitoba, Winnipeg, Manitoba, Canada; 33 Department of Pediatrics and Child Health, University of Manitoba, Winnipeg, Manitoba, Canada; 34 Department of Emergency Medicine, Montefiore-Nyack Hospital, Nyack, New York; 35 Division of Pediatric Emergency Medicine, Department of Pediatrics, University of Alabama at Birmingham; 36 University of Alberta, Stollery Children’s Hospital, Women’s and Children’s Health Research Institute, Edmonton, Alberta, Canada; 37 UPMC Children’s Hospital of Pittsburgh, Pittsburgh, Pennsylvania; 38 Primary Children’s Hospital, Intermountain Healthcare, Salt Lake City, Utah; 39 Department of Emergency Medicine, University of Michigan School of Medicine, Ann Arbor; 40 Department of Pediatrics, University of Michigan School of Medicine, Ann Arbor; 41 Departamento de Emergencia Pediátrica, Hospital General Pediátrico Niños de Acosta Ñu, Facultad de Medicina, Universidad Privada del Pacífico, San Lorenzo, Paraguay; 42 Children’s Emergency Department, Starship Children’s Hospital, Auckland, New Zealand; 43 Department of Surgery, University of Auckland, Auckland, New Zealand; 44 Department of Paediatrics: Child and Youth Health, University of Auckland, Auckland, New Zealand; 45 Centers for Disease Control and Prevention, Atlanta, Georgia; 46 Division of Infectious Diseases, Boston Children’s Hospital and Harvard Medical School, Boston, Massachusetts; 47 Emergency Department, Perth Children’s Hospital, Perth, Western Australia; 48 Division of Emergency Medicine, School of Medicine, University of Western Australia, Perth, Australia; 49 Division of Paediatrics, School of Medicine, University of Western Australia, Perth, Australia; 50 Division of Emergency Medicine, Department of Pediatrics, Medical College of Wisconsin, Milwaukee; 51 Department of Pediatrics, Children’s Hospital of Eastern Ontario, Ottawa, Ontario, Canada; 52 Hospital de Pediatría “Prof Dr. Juan P. Garrahan,” RIDEPLA, Buenos Aires, Argentina; 53 Department of Pediatrics, Hospital Francesc de Borja, Gandia, Spain; 54 Department of Pediatrics, University of Cincinnati College of Medicine, Cincinnati, Ohio; 55 Division of Pediatric Emergency Medicine, Cincinnati Children’s Hospital, Cincinnati, Ohio; 56 Department of Pediatrics, Division of Emergency Medicine, McMaster Children’s Hospital, Hamilton, Ontario, Canada; 57 Department of Emergency Medicine, KK Women’s and Children’s Hospital, Duke-NUS Medical School, SingHealth Duke-NUS Global Health Institute, Singapore; 58 ASST Spedali Civili di Brescia–Pronto soccorso pediatrico, Brescia, Italy; 59 Department of Emergency Medicine, New York Presbyterian Morgan Stanley Children’s Hospital, Columbia University Irving Medical Center, New York, New York; 60 Department of Pediatric Emergency Medicine, IWK Health Centre, Dalhousie University, Halifax, Nova Scotia, Canada; 61 Department of Pediatrics, Schulich School of Medicine & Dentistry, London, Ontario, Canada; 62 Department of Emergency Medicine, Lincoln Medical Center, New York, New York; 63 Data Analytics and Reporting, Ann and Robert H. Lurie Children’s Hospital of Chicago, Chicago, Illinois; 64 Section of Pediatric Emergency Medicine, Department of Pediatrics, Cumming School of Medicine, University of Calgary, Calgary, Alberta, Canada; 65 Section of Gastroenterology, Department of Pediatrics, Cumming School of Medicine, University of Calgary, Calgary, Alberta, Canada; 66 Department of Emergency Medicine, Cumming School of Medicine, University of Calgary, Calgary, Alberta, Canada

## Abstract

**Importance:**

Little is known about the risk factors for, and the risk of, developing post–COVID-19 conditions (PCCs) among children.

**Objectives:**

To estimate the proportion of SARS-CoV-2–positive children with PCCs 90 days after a positive test result, to compare this proportion with SARS-CoV-2–negative children, and to assess factors associated with PCCs.

**Design, Setting, and Participants:**

This prospective cohort study, conducted in 36 emergency departments (EDs) in 8 countries between March 7, 2020, and January 20, 2021, included 1884 SARS-CoV-2–positive children who completed 90-day follow-up; 1686 of these children were frequency matched by hospitalization status, country, and recruitment date with 1701 SARS-CoV-2–negative controls.

**Exposure:**

SARS-CoV-2 detected via nucleic acid testing.

**Main Outcomes and Measures:**

Post–COVID-19 conditions, defined as any persistent, new, or recurrent health problems reported in the 90-day follow-up survey.

**Results:**

Of 8642 enrolled children, 2368 (27.4%) were SARS-CoV-2 positive, among whom 2365 (99.9%) had index ED visit disposition data available; among the 1884 children (79.7%) who completed follow-up, the median age was 3 years (IQR, 0-10 years) and 994 (52.8%) were boys. A total of 110 SARS-CoV-2–positive children (5.8%; 95% CI, 4.8%-7.0%) reported PCCs, including 44 of 447 children (9.8%; 95% CI, 7.4%-13.0%) hospitalized during the acute illness and 66 of 1437 children (4.6%; 95% CI, 3.6%-5.8%) not hospitalized during the acute illness (difference, 5.3%; 95% CI, 2.5%-8.5%). Among SARS-CoV-2–positive children, the most common symptom was fatigue or weakness (21 [1.1%]). Characteristics associated with reporting at least 1 PCC at 90 days included being hospitalized 48 hours or more compared with no hospitalization (adjusted odds ratio [aOR], 2.67 [95% CI, 1.63-4.38]); having 4 or more symptoms reported at the index ED visit compared with 1 to 3 symptoms (4-6 symptoms: aOR, 2.35 [95% CI, 1.28-4.31]; ≥7 symptoms: aOR, 4.59 [95% CI, 2.50-8.44]); and being 14 years of age or older compared with younger than 1 year (aOR, 2.67 [95% CI, 1.43-4.99]). SARS-CoV-2–positive children were more likely to report PCCs at 90 days compared with those who tested negative, both among those who were not hospitalized (55 of 1295 [4.2%; 95% CI, 3.2%-5.5%] vs 35 of 1321 [2.7%; 95% CI, 1.9%-3.7%]; difference, 1.6% [95% CI, 0.2%-3.0%]) and those who were hospitalized (40 of 391 [10.2%; 95% CI, 7.4%-13.7%] vs 19 of 380 [5.0%; 95% CI, 3.0%-7.7%]; difference, 5.2% [95% CI, 1.5%-9.1%]). In addition, SARS-CoV-2 positivity was associated with reporting PCCs 90 days after the index ED visit (aOR, 1.63 [95% CI, 1.14-2.35]), specifically systemic health problems (eg, fatigue, weakness, fever; aOR, 2.44 [95% CI, 1.19-5.00]).

**Conclusions and Relevance:**

In this cohort study, SARS-CoV-2 infection was associated with reporting PCCs at 90 days in children. Guidance and follow-up are particularly necessary for hospitalized children who have numerous acute symptoms and are older.

## Key Points

**Question:**

What proportion of children infected with SARS-CoV-2 who were tested in emergency departments (EDs) reported post–COVID-19 conditions (PCCs) 90 days after their ED visits?

**Findings:**

In this cohort study of 1884 SARS-CoV-2–positive children with 90-day follow-up, 5.8% of patients, including 9.8% of hospitalized children and 4.6% of discharged children, reported PCCs. Characteristics associated with PCCs included being hospitalized 48 hours or more, having 4 or more symptoms reported at the index ED visit, and being 14 years of age or older.

**Meaning:**

This study suggests that, given the prevalence of PCCs, appropriate guidance and follow-up are required for children testing positive for SARS-CoV-2.

## Teaser

This cohort study estimates the proportion of SARS-CoV-2–positive children with post–COVID-19 conditions 90 days after a positive test result, compares this proportion with SARS-CoV-2–negative children, and assesses factors associated with post–COVID-19 conditions.

## Introduction

Persistent, new, or returning health problems (post–COVID-19 conditions [PCCs]) may occur after SARS-CoV-2 infections.^1,2^ Although PCCs have been described primarily in adults,^3,4^ concern regarding PCCs in children has been growing.^5^ Even among adults, clinicians must be cautious when attributing PCCs after COVID-19 to SARS-CoV-2, as research has more strongly associated persistent physical symptoms with self-reported, but serology-negative, COVID-19 infection rather than laboratory-confirmed infection.^6^

Post–COVID-19 conditions among children remain poorly described. Early reports estimated that 25% to 58% of children experienced PCCs months after their acute illnesses and occurrence was not associated with disease severity.^7,8,9^ However, a subsequent study that included primarily nonhospitalized, SARS-CoV-2–positive children reported that only 4% were symptomatic 28 days after being tested and 2% were symptomatic 56 days after being tested.^10^ Although these risks were higher than reported among noninfected participants, other reports^11,12^ described no difference in the frequency of PCCs among pediatric COVID-19 patients and controls. Limitations of these reports include testing criteria that relied on typical adult symptoms, low follow-up rates,^10^ reliance on health care encounter information,^11^ small sample sizes, and antibody-based exposure classification.^12^

A comprehensive understanding of pediatric PCCs is required to inform public health policies and guide the care of high-risk children.^13^ Therefore, we identified the proportion of children with PCCs 90 days after SARS-CoV-2 testing, stratified by hospitalization status, in a prospective multinational pediatric cohort. We identified risk factors for PCCs among SARS-CoV-2–positive children and compared the prevalence of PCCs among this group with a matched cohort of SARS-CoV-2–negative children.

## Methods

### Design

Participants were recruited in 39 pediatric emergency departments (EDs) in the Pediatric Emergency Research Network^14^–COVID-19 Study between March 7, 2020, and January 20, 2021. The final cohort comprised children from 36 of the 39 EDs. Participants were enrolled in 8 countries (Argentina, Canada, Costa Rica, Italy, Paraguay, Singapore, Spain, and the United States).^15^ Participating sites had local ethics review board approval or established a reliance agreement with the Cincinnati Children’s Hospital Medical Center institutional review board. Legal guardians of all participants provided informed consent (written or verbal based on local ethics requirements) and children provided assent, as appropriate. This study followed the Strengthening the Reporting of Observational Studies in Epidemiology (STROBE) reporting guideline.^16^

### Participants

Children younger than 18 years who underwent testing for SARS-CoV-2 at participating EDs because of symptoms or epidemiologic risk factors (eg, close contact of a case) were eligible. Initially, participants were enrolled consecutively, based on timing of test performance, regardless of SARS-CoV-2 test result, up to a maximum of 5 enrollments per day per site. In regions with low positivity rates, this led to overenrollment of SARS-CoV-2–negative participants. Therefore, after September 2020, sites consecutively enrolled as many SARS-CoV-2–positive participants as possible, along with 2 consecutive participants who tested negative for every participant enrolled who tested positive.

### Objective

The primary objective was to assess the proportion of SARS-CoV-2–positive participants with PCCs, stratified by hospitalization status, 90 days after the index ED visit. We sought to identify risk factors for PCCs among SARS-CoV-2–positive children. To better understand the association between PCCs and SARS-CoV-2 infection, we compared PCCs between infected children and matched children who tested negative.

### Data Collection

Demographic and medical information was collected via caregiver interviews. Follow-up telephone (or email or text, depending on site) surveys were completed 14 days after the index ED visit to classify outcomes. Medical record reviews were performed to confirm caregiver-reported index ED visit disposition and 14-day outcome data. Between 90 and 120 days after the index ED visit, caregivers were contacted and asked if their child had any persistent, new, or returning symptoms or health problems that may have been associated with the illness prompting the initial ED evaluation.

### Definitions

SARS-CoV-2 status was classified as positive if a nucleic acid test performed on a swab sample obtained from the nares, nasopharynx, or oral cavity was positive at the index ED visit or during the subsequent 14 days. Participants with negative nucleic acid tests constituted the comparison group. Acute symptoms were those present between symptom onset and the time of the index ED visit. Acute SARS-CoV-2 illness hospitalization and illness severity status incorporated events occurring until 14 days after the index ED visit. Illness severity was classified as severe or not severe based on specific interventions (eg, positive pressure ventilation, inotropic support), the occurrence of specific complications, organ dysfunction, or death (eAppendix in Supplement 1). Participants were considered lost to follow-up if 5 follow-up attempts were unsuccessful.

Post–COVID-19 conditions were present if the caregiver indicated at the 90-day interview that the participant had any persistent, new, or returning symptoms or health problems.^2^ Post–COVID-19 conditions were not present if the caregiver indicated that these symptoms were neither persistent (ie, recovered completely prior to 90 days) nor novel (ie, underlying condition without exacerbation). Post–COVID-19 conditions were classified as cardiovascular, dermatologic, ophthalmologic or otolaryngologic, gastrointestinal, neurologic, psychological, respiratory, systemic (eg, fatigue, weakness, fever, anorexia), or other. Caregivers could indicate the presence of PCCs using check boxes or free text. For the latter, 1 author (A.L.F.) blinded to SARS-CoV-2 test status performed narrative review and grouping. The PCC term also reflected health problems reported by children who tested negative, to permit comparisons.

### Sample Size

Specification for the Pediatric Emergency Research Network–COVID-19 prospective cohort study included the recruitment of up to 12 500 participants to enroll the 50 or more COVID-19–positive children who experienced the study’s primary objective of a severe outcome.^15^ When the parent study achieved the desired number of children,^17^ recruitment was terminated. During the planning of the parent study, we anticipated that our reporting of the proportion of SARS-CoV-2–positive participants with PCCs would have sufficient precision using the achieved cohort, as reflected by 95% CIs.^18^

### Statistical Analysis

Data are presented as counts and percentages, mean (SD) values, and median (IQR) values. Categorical variables were compared using the Fisher exact test or the χ^2^ test, as appropriate. Median values were compared using the Wilcoxon rank-sum test. The Cochran-Armitage test was used to assess the linear trend in PCCs associated with the number of symptoms at the index ED visit.

The primary outcome, PCCs, was stratified by hospitalization status. Owing to the small numbers of SARS-CoV-2–positive participants in some countries, to permit analyses to incorporate a variable to represent a participant’s country, those recruited in Argentina (n = 13), Italy (n = 1), Paraguay (n = 28), and Singapore (n = 2) were combined into a single grouping; all other countries were analyzed independently. We used multiple logistic regression to identify factors associated with PCCs among SARS-CoV-2–positive patients. Variables included in the model were country of enrollment, sex, age, chronic underlying condition (excluding asthma), number of symptoms at the index ED visit (categorized as 0, 1-3, 4-6, or ≥7, with cut points selected to evenly distribute participants across categories), hospitalization as a 3-level categorical variable incorporating length of stay (none, <48 hours, or ≥48 hours), and month of enrollment. We used least absolute shrinkage and selection operator via 10-fold cross-validation with 100 lambdas for variable selection. We then fit a multiple logistic regression model with the variables selected by least absolute shrinkage and selection operator to obtain the adjusted odds ratio (aOR) associated with PCCs.

In unadjusted analyses, we frequency-matched SARS-CoV-2–positive and SARS-CoV-2–negative participants by randomly selecting an equal number of SARS-CoV-2–positive and SARS-CoV-2–negative participants enrolled within each country within 1 calendar month. We used multiple logistic regression to identify factors associated with PCCs in the frequency-matched subsample, following the same model selection strategy as described for the SARS-CoV-2–positive children, but with the inclusion of an indicator for SARS-CoV-2 positivity as a covariate. For each specific persistent symptom present among 20 or more members of the frequency-matched subsample, a separate multiple logistic regression model was fit to obtain the aOR associated with SARS-CoV-2 positivity. All models included country and hospitalization as 5-level and 3-level categorical variables, respectively.

We report the number of missing data values for key variables. When computing symptom counts as independent variables, missing values were treated as absent.^19^ When PCCs were the dependent variable, we did not impute missing values, as the missing-at-random assumption was not thought to be met in this scenario. All statistical analyses were 2-sided and performed using Stata, version 16 (StataCorp LLC), with significance defined by a *P* < .05, with adjustment for multiplicity of comparisons performed using the Benjamini-Hochberg approach.^20^

## Results

**Figure 1.**
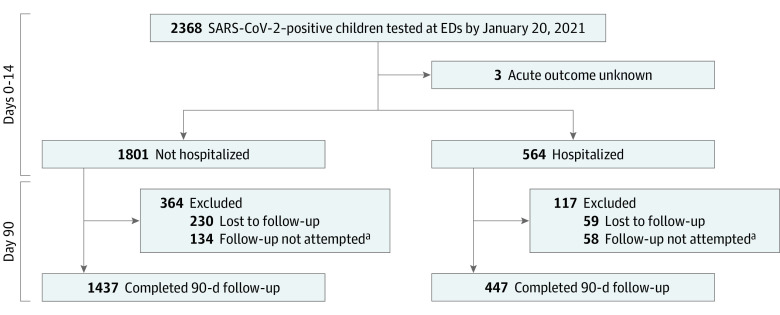
Flowchart of SARS-CoV-2–Positive Participants, Including Follow-up ED indicates emergency department. ^a^In a few sites, consistent 90-day follow-up was not feasible amid human resource constraints during the COVID-19 pandemic.

A total of 8642 children were enrolled, of whom 2368 (27.4%) were SARS-CoV-2 positive; 2365 of these 2368 children (99.9%) had disposition data available. A total of 1884 of 2365 children (79.7%) completed 90-day follow-up (Figure 1). The median age of the 1884 participants was 3 years (IQR, 0-10 years), 994 (52.8%) were boys, and the most common ED index visit symptoms were fever (1241 [65.9%]), cough (917 [48.7%]), and rhinorrhea or congestion (893 [47.4%]) (Table 1). Children lost to follow-up differed from those with complete follow-up in terms of country and number of index ED visit symptoms (eTable 1 in Supplement 1).

**Table 1.** Baseline Characteristics of 1884 SARS-CoV-2–Positive Children With Complete Follow-up Based on Hospitalization Status

Characteristic	Children, No. (%)		
	All (N = 1884)	Acute (days 0-14) SARS-CoV-2 outcome	
		Not hospitalized (n = 1437)	Hospitalized (n = 447)
Region			
United States	1204 (63.9)	927 (64.5)	277 (62.0)
Costa Rica	331 (17.6)	232 (16.1)	99 (22.2)
Canada	172 (9.1)	158 (11.0)	14 (3.1)
Spain	133 (7.1)	101 (7.0)	32 (7.2)
Other^a^	44 (2.3)	19 (1.3)	25 (5.6)
Sex			
Male	994 (52.8)	748 (52.1)	246 (55.0)
Female	890 (47.2)	689 (48.0)	201 (45.0)
Age, y			
Median (IQR)	3 (0-10)	3 (0-10)	4 (0-12)
<1.0	490 (26.0)	373 (26.0)	117 (26.2)
1.0 to <2.0	232 (12.3)	191 (13.3)	41 (9.2)
2.0 to <5.0	295 (15.7)	221 (15.4)	74 (16.6)
5.0 to <10.0	364 (19.3)	289 (20.1)	75 (16.8)
10.0 to <14.0	240 (12.7)	181 (12.6)	59 (13.2)
14.0 to <18.0	263 (14.0)	182 (12.7)	81 (18.1)
Chronic underlying condition^b^	270 (14.3)	155 (10.8)	115 (25.7)
History of asthma^b^	260 (13.8)	194 (13.5)	66 (14.8)
No. of symptoms at ED presentation			
Median (IQR)	4 (2-6)	4 (2-6)	4 (2-6)
None	113 (6.0)	80 (5.6)	33 (7.4)
1-3	754 (40.0)	585 (40.7)	169 (37.8)
4-6	626 (33.2)	478 (33.3)	148 (33.1)
≥7	391 (20.8)	294 (20.5)	97 (21.7)
Types of symptoms^c^			
Any systemic symptom	1519 (80.6)	1153 (80.2)	366 (81.9)
Fever	1241 (65.9)	950 (66.1)	291 (65.1)
Drowsy or lethargic	624 (33.1)	479 (33.3)	145 (32.4)
Irritability	526 (27.9)	405 (28.2)	121 (27.1)
Anorexia	492 (26.1)	363 (25.3)	129 (28.9)
Myalgia	290 (15.4)	225 (15.7)	65 (14.5)
Arthralgia	185 (9.8)	137 (9.5)	48 (10.7)
Edema of extremities^d^	34 (1.8)	19 (1.3)	15 (3.4)
Any respiratory symptom	1328 (70.5)	1073 (74.7)	255 (57.1)
Cough	917 (48.7)	758 (52.8)	159 (35.6)
Runny nose or congestion	893 (47.4)	735 (51.2)	158 (35.4)
Sore throat	341 (18.1)	291 (20.3)	50 (11.2)
Difficulty breathing	311 (16.5)	216 (15.0)	95 (21.3)
Chest pain^e^	126 (6.7)	95 (6.6)	31 (6.9)
Wheezing	122 (6.5)	85 (5.9)	37 (8.3)
Other respiratory symptoms^f^	35 (1.9)	21 (1.5)	14 (3.1)
Any gastrointestinal symptom	749 (39.8)	545 (37.9)	204 (45.6)
Diarrhea	393 (20.9)	291 (20.3)	102 (22.8)
Vomiting	359 (19.1)	236 (16.4)	123 (27.5)
Abdominal pain^g^	343 (18.2)	225 (15.7)	118 (26.4)
Any neurologic symptom	538 (28.6)	415 (28.9)	123 (27.5)
Headache	461 (24.5)	371 (25.8)	90 (20.1)
Loss of smell or taste^h^	111 (5.9)	91 (6.3)	20 (4.5)
Seizures	53 (2.8)	19 (1.3)	34 (7.6)
Rash (general, hand, or foot)	204 (10.8)	148 (10.3)	56 (12.5)
Conjunctivitis	121 (6.4)	85 (5.9)	36 (8.1)
Oral symptoms^d^	83 (4.4)	60 (4.2)	23 (5.2)

Abbreviation: ED, emergency department.

^a^ Participants from Argentina (n = 13), Italy (n = 1), Paraguay (n = 28), and Singapore (n = 2) were combined owing to the small numbers of SARS-CoV-2–positive participants from each country.

^b^ Information was missing for 1 child who was not hospitalized during the acute phase of illness.

^c^ Information was missing for a maximum of 3 children for each specific symptom type, unless otherwise noted; those with greater amounts of missing data are due to database modifications that occurred on August 20, 2020, to capture symptoms associated with multisystem inflammatory syndrome of children.

^d^ Including redness or sores in the mouth; information missing for 557 participants (406 not hospitalized, 151 hospitalized).

^e^ Information missing for 603 participants (468 not hospitalized, 135 hospitalized).

^f^ Includes apnea and sputum production; information missing for 126 participants (88 not hospitalized, 38 hospitalized).

^g^ Among the children with abdominal pain, 24 (7.0%) had multisystem inflammatory syndrome of children; 7 of 31 children (22.6%) with multisystem inflammatory syndrome of children did not have abdominal pain.

^h^ Information missing for 558 participants (406 not hospitalized, 152 hospitalized).

### Outcomes for SARS-CoV-2–Positive Participants

A total of 110 SARS-CoV-2–positive children (5.8% [95% CI, 4.8%-7.0%]) reported 90-day PCCs (eTable 2 in Supplement 1). This rate was higher among hospitalized children (44 of 447 [9.8%; 95% CI, 7.2%-13.0%]), regardless of symptom severity, compared with those discharged from the ED (66 of 1437 [4.6%; 95% CI, 3.6%-5.8%]; difference, 5.3% [95% CI, 2.5%-8.5%]). Among hospitalized children, those who experienced severe outcomes within 14 days were more likely to report 90-day PCCs than those who did not (13 of 70 [18.6%; 95% CI, 10.3%-29.7%] vs 31 of 377 [8.2%; 95% CI, 5.7%-11.5%]; difference, 10.4% [95% CI, 2.3%-21.3%]).

Most children reporting PCCs at 90 days had 1 persistent, new, or recurring health problem (59.1% [65 of 110]) (eTable 3 in Supplement 1). The most common PCC symptoms were respiratory (38 of 1884 [2.0%; 95% CI, 1.4%-2.8%]) and systemic (33 of 1884 [1.8%; 95% CI, 1.2%-2.5%]). Fatigue or weakness was the most reported individual symptom (21 of 1884 [1.1%]). A total of 24 of 45 participants (53.3%) reported more than 1 new PCC, including at least 1 systemic symptom (eFigure 1 in Supplement 1). Among hospitalized and nonhospitalized children, the proportion of children reporting PCCs was higher among children with a greater number of index ED visit symptoms (hospitalized children: no symptoms, 3 of 33 [9.1%]; 1-3 symptoms, 8 of 169 [4.7%]; 4-6 symptoms, 11 of 148 [7.4%]; and ≥7 symptoms, 22 of 97 [22.7%]; and nonhospitalized children: no symptoms, 1 of 80 [1.3%]; 1-3 symptoms, 9 of 585 [1.5%]; 4-6 symptoms, 23 of 478 [4.8%]; and ≥7 symptoms, 33 of 294 [11.2%]; Cochran-Armitage test: *P* < .001 for trend) (eTable 2 in Supplement 1).

### Adjusted Analysis of SARS-CoV-2–Positive Participants

Characteristics associated with PCCs at 90-day follow-up included being hospitalized 48 hours or more compared with no hospitalization (aOR, 2.67 [95% CI, 1.63-4.38]), having 4 or more symptoms reported at the index ED visit compared with 1 to 3 symptoms (4-6 symptoms: aOR, 2.35 [95% CI, 1.28-4.31]; ≥7 symptoms: aOR, 4.59 [95% CI, 2.50-8.44]), and being 14 years of age or older compared with younger than 1 year (aOR, 2.67 [95% CI, 1.43-4.99]) (Table 2).

**Table 2.** Multiple Logistic Regression Model Demonstrating Factors Associated With Reporting of Persistent, New, or Recurring Health Problem in 1875 SARS-CoV-2–Positive Children With Complete Data^a^

Factor	No./total No.	aOR (95% CI)	*P* value
Region			
United States	79/1200	1 [Reference]	NA
Costa Rica	10/329	0.70 (0.33-1.46)	.34
Canada	16/170	1.61 (0.87-2.98)	.13
Spain	3/133	0.60 (0.18-2.01)	.41
Other^b^	0/43	Excluded	NA
Sex			
Male	51/987	1 [Reference]	NA
Female	57/888	1.38 (0.92-2.08)	.12
Age, y			
<1.0	19/488	1 [Reference]	NA
1.0 to <2.0	7/231	0.84 (0.34-2.06)	.71
2.0 to <5.0	9/291	0.84 (0.37-1.92)	.68
5.0 to <10.0	19/364	1.40 (0.71-2.75)	.33
10.0 to <14.0	20/238	1.91 (0.97-3.76)	.06
14.0 to <18.0	34/263	2.67 (1.43-4.99)	.002
Chronic condition (other than asthma)			
No	85 1065	1 [Reference]	NA
Yes	23/269	1.04 (0.62-1.76)	.88
No. of symptoms at ED presentation			
Asymptomatic	4/111	1.35 (0.44-4.19)	.60
1-3	17/752	1 [Reference]	NA
4-6	34/624	2.35 (1.28-4.31)	.006
≥7	55/388	4.59 (2.50-8.44)	<.001
Hospitalized for acute illness			
No	66/1437	1 [Reference]	NA
Yes, <48 h	10/148	2.07 (0.99-4.32)	.05
Yes, ≥48 h	32/290	2.67 (1.63-4.38)	<.001
Season of infection			
Spring 2020 (Mar-May)	6/186	0.47 (0.19-1.18)	.11
Summer 2020 (Jun-Aug)	30/696	1 [Reference]	NA
Fall 2020 (Sep-Nov)	41/616	1.25 (0.74-2.09)	.41
Winter 2020-2021 (Dec-Jan)	31/377	1.22 (0.69-2.14)	.50

Abbreviations: aOR, adjusted odds ratio; ED, emergency department; NA, not applicable.

^a^ Covariates included in the final model were selected using least absolute shrinkage and selection operator for assessment.

^b^ Participants from Argentina (n = 13), Italy (n = 1), Paraguay (n = 28), and Singapore (n = 2) were combined owing to the small numbers of SARS-CoV-2–positive participants from each country.

### Comparisons With SARS-CoV-2–Negative Participants

Frequency matching was possible for 474 hospitalized and 1626 nonhospitalized pairs of SARS-CoV-2–positive and SARS-CoV-2–negative children (eFigures 2 and 3 and eTable 4 in Supplement 1). Among children discharged from the ED, PCCs were reported by 55 of 1295 SARS-CoV-2–positive children (4.2% [95% CI, 3.2%-5.5%]) and 35 of 1321 SARS-CoV-2–negative children (2.7% [95% CI, 1.9%-3.7%]; difference, 1.6% [95% CI, 0.2%-3.0%]) (Table 3). Among hospitalized children, PCCs were reported at 90 days in 40 of 391 SARS-CoV-2–positive children (10.2% [95% CI, 7.4%-13.7%]) and 19 of 380 SARS-CoV-2–negative children (5.0% [95% CI, 3.0%-7.7%]; difference, 5.2% [95% CI, 1.5%-9.1%]). Rates of PCCs were greater among SARS-CoV-2–positive children in several demographic and clinical strata (eTable 5 in Supplement 1).

**Table 3.** Comparison of Any Reported Persistent, New, or Recurring Health Problems 90 Days After the Index Visit Among Frequency Matched SARS-CoV-2–Positive and SARS-CoV-2–Negative Children Who Were Symptomatic at the Time of Testing

Factor	Not hospitalized (days 0-14)			Hospitalized (days 0-14)		
	SARS-CoV-2 positive	SARS-CoV-2 negative	Adjusted *P* value^a^	SARS-CoV-2 positive	SARS-CoV-2 negative	Adjusted *P* value^a^
All children, No./total No. (%) [95% CI]	55/1295 (4.2) [3.2-5.5]	35/1321 (2.7) [1.9-3.7]	.14	40/391 (10.2) [7.4-13.7]	19/380 (5.0) [3.0-7.7]	.03
Age group, No./total No. (%)						
<1.0 y	8/338 (2.4)	6/258 (2.3)	>.99	6/99 (6.1)	4/84 (4.8)	.96
1.0 to <2.0 y	4/173 (2.3)	8/265 (3.0)	>.99	1/34 (2.9)	0/41 (0)	.67
2.0 to <5.0 y	3/198 (1.5)	6/312 (1.9)	>.99	4/60 (6.7)	2/73 (2.7)	.67
5.0 to <10.0 y	16/260 (6.2)	6/246 (2.4)	.14	3/69 (4.4)	4/70 (5.7)	>.99
10.0 to <14.0 y	10/164 (6.1)	2/133 (1.5)	.14	8/49 (16.3)	5/62 (8.1)	.47
14.0 to <18.0 y	14/162 (8.6)	7/107 (6.5)	.97	18/80 (22.5)	4/50 (8.0)	.09
Chronic condition, No./total No. (%)						
Yes	8/131 (6.1)	8/215 (3.7)	.53	11/104 (10.6)	9/103 (8.7)	.96
No	47/1163 (4.0)	27/1106 (2.4)	.14	29/287 (10.1)	10/277 (3.6)	.01
No. of acute symptoms, No./total No. (%)^b^						
1-3	7/513 (1.4)	6/472 (1.3)	>.99	7/149 (4.7)	7/153 (4.6)	>.99
4-6	20/442 (4.5)	11/475 (2.3)	.14	11/128 (8.6)	8/139 (5.8)	.67
≥7	27/267 (10.1)	16/277 (5.8)	.14	21/90 (23.3)	3/64 (4.7)	.01
Severe acute illness, No./total No. (%)						
Yes	NA	NA	NA	12/67 (17.9)	1/50 (2.0)	.03
No	NA	NA	NA	28/324 (8.6)	18/330 (5.5)	.26

Abbreviation: NA, not applicable.

^a^ Reported *P* values have been converted within groups (ie, not hospitalized or hospitalized) to adjusted *P* values (*q* values), using the Benjamini-Hochberg method to account for multiple comparisons.

^b^ Cochran-Armitage test for trend among nonhospitalized, SARS-CoV-2–positive and SARS-CoV-2–negative children was less than .001 and .003, respectively. Among hospitalized, SARS-CoV-2–positive and SARS-CoV-2–negative children, the Cochran-Armitage test for trend was less than .001 and .98, respectively.

**Figure 2.**
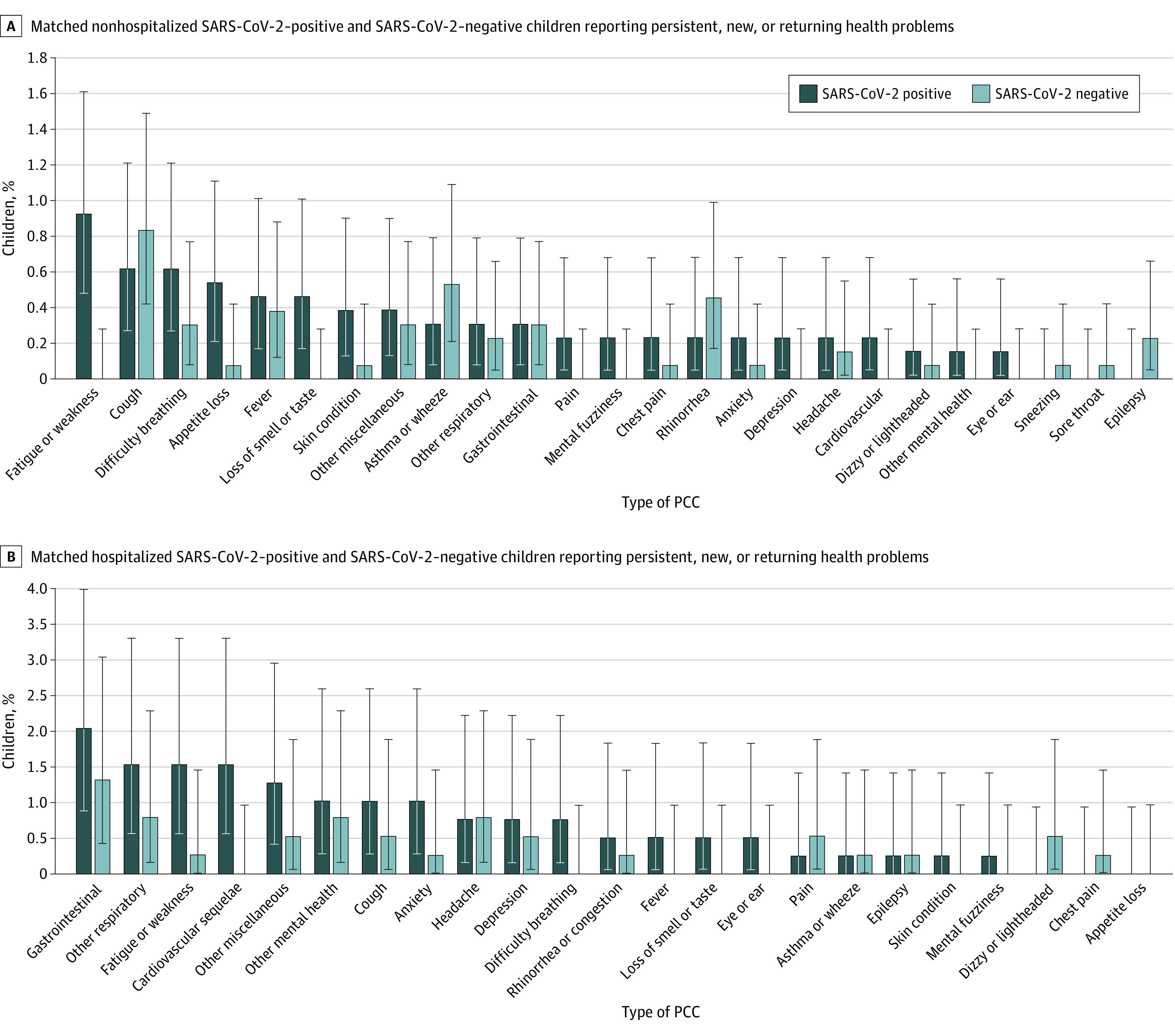
Percentage of Frequency-Matched Nonhospitalized and Hospitalized Children A, Matched nonhospitalized SARS-CoV-2–positive and SARS-CoV-2–negative children reporting persistent, new, or recurring health problems. B, Matched hospitalized SARS-CoV-2–positive and SARS-CoV-2–negative children reporting persistent, new, or recurring health problems. The whiskers indicate 95% CIs around the proportion point estimate, indicated by the height of the bar. PCC indicates post–COVID-19 condition.

Among children not hospitalized, systemic (primarily fatigue or weakness) symptoms (21 of 1295 [1.6%] vs 6 of 1321 [0.5%]; difference, 1.2% [95% CI, 0.4%-2.1%]), psychological symptoms (9 of 1295 [0.7%] vs 2 of 1321 [0.2%]; difference, 0.5% [95% CI, 0.03%-1.2%]), and anosmia or ageusia (6 of 1295 [0.5%] vs 0 of 1321; difference, 0.5% [95% CI, 0.08%-1.0%]) were reported more frequently by SARS-CoV-2–positive vs SARS-CoV-2–negative children (Figure 2A; eTable 5 in Supplement 1). Among hospitalized children, only cardiovascular PCCs (6 of 391 [1.5%] vs 0 of 380; difference, 1.5% [95% CI, 0.2%-3.3%]) were more common among SARS-CoV-2–positive children (Figure 2B; eTable 5 in Supplement 1). In adjusted analysis, any PCC reported at 90 days (aOR, 1.63 [95% CI, 1.14-2.35]), and specifically systemic health problems (eg, fatigue, weakness, fever; aOR, 2.44 [95% CI, 1.19-5.00]), were associated with SARS-CoV-2 infection (eTable 6 in Supplement 1).

## Discussion

This study found that 9.8% of hospitalized children with SARS-CoV-2 infections and 4.6% of nonhospitalized children with SARS-CoV-2 infections reported PCCs at 90 days; similar symptoms were reported by 5.0% of matched hospitalized children without SARS-CoV-2 infections and 2.7% of matched nonhospitalized children without SARS-CoV-2 infections. The most common PCC symptoms among SARS-CoV-2–infected children were respiratory (eg, cough, difficulty breathing, or shortness of breath; 2.0%) and systemic (eg, fatigue or weakness; 1.8%). Risk factors associated with PCCs included length of hospitalization, higher number of symptoms at the index ED visit, and older age. The most common PCCs were fatigue and weakness.

Our estimated 90-day PCC prevalence among children infected with SARS-CoV-2 is lower than in earlier reports describing small cohorts of hospitalized children.^9,21,22^ The largest early report was a prospective study that included 518 Russian children, among whom 25% reported PCCs after more than 5 months.^9^ In our study, only 9.8% of hospitalized children (44 of 447) experienced PCCs at 90 days. The lower PCC prevalence in our study may reflect a higher follow-up rate and thus lower risk of bias and our use of different approaches to eliciting PCCs.

Early pediatric outpatient studies also reported a high PCC prevalence,^23^ including a cohort study of primarily asymptomatic children that described PCCs at 3 months in 66% of SARS-CoV-2–positive participants and 53% of SARS-CoV-2–negative participants.^24^ These estimates are likely inflated by self-selection bias, as the follow-up rate was only 13%. Subsequent studies reported lower frequencies of PCCs in children.^25,26,27^ Among 97 symptomatic infected children in Australia, 12% reported PCCs at 3- to 6-month follow-up^25^; among 175 children who completed a weekly symptom diary in the United Kingdom, 5% reported persistent symptoms.^26^ Last, among 1734 SARS-CoV-2–positive children in the United Kingdom who completed a symptom diary, only 4% had an illness duration of 28 days or longer.^10^ The latter study included control children with negative SARS-CoV-2 test results, of whom 0.9% reported ongoing symptoms at 28 days. The differing estimates provided by the aforementioned studies likely reflect variations in response rates, PCC definitions, and approaches to identifying PCCs.^10^ Nonetheless, they all suggest an increased prevalence of PCCs among SARS-CoV-2–infected children compared with uninfected children.

The most commonly reported persistent symptom in our SARS-CoV-2–positive participants was fatigue. Although other studies have identified that fatigue, headache, and anosmia are common PCCs in children,^10,26^ fatigue is the most common PCC symptom reported in adults.^28,29,30^ Anosmia, which was uncommon in our cohort, was a predominant symptom among children in a large UK study, where it was a core symptom determining access to testing during the study period.^10^ Thus, local testing criteria can influence PCC symptoms.

Our finding of an increased risk of PCCs among SARS-CoV-2–positive children compared with SARS-CoV-2–negative children provides important and generalizable evidence. This finding contradicts those provided by a matched administrative database analysis,^11^ likely reflecting differences in outcome measures (ie, self-reported symptoms vs diagnosed conditions) and data ascertainment methods. Moreover, the administrative database study included few children and SARS-CoV-2 test results were not accessed to classify disease status.

Previously identified pediatric PCC risk factors include hospitalization,^9^ female sex,^26^ and a history of allergic^9^ and underlying chronic diseases.^26^ In keeping with earlier reports,^9,10^ we identified that older age was associated with reporting of PCCs. This finding may reflect the fact that younger, less-verbal children are less likely to report specific symptoms, compared with verbal teenagers. In addition, we identified hospitalization and 4 or more acute symptoms as risk factors associated with 90-day PCCs. Although the latter finding may reflect the possibility that individuals reporting more symptoms at baseline are inherently more likely to report symptoms in general, it is consistent with research in adults that found that the presence of 5 or more symptoms during the acute illness was associated with PCCs.^28^

### Strengths and Limitations

To our knowledge, this large cohort study is the first to explore PCCs among children seeking ED care. Strengths include a SARS-CoV-2–negative control group, prospective data collection, and consistent and identical follow-up for all participants. Our PCC assessment approach, which clarifies the presence of the reported symptoms prior to the index ED visit, represents a methodological advance compared with prior assessments that focused on the presence or absence of symptoms in the preceding 7 days.^12^ We recruited children in numerous countries and captured repeated SARS-CoV-2 test results within 14 days to appropriately classify children with false-negative index test results.^31^ Thus, our evaluation provides a realistic estimate of PCC prevalence along with a comparator group. Although a nonnegligible proportion of SARS-CoV-2–negative children reported symptoms that met our definition of PCCs, persistent, new, or recurring health problems were more common among SARS-CoV-2–positive children. In adjusted analyses, reporting PCCs was associated with a positive SARS-CoV-2 test result. Nonetheless, we cannot exclude the possibility that this association is not specific to SARS-CoV-2 infection and may reflect the association that infection has with perception of symptoms.^6^

This study also has some limitations. We defined the presence of PCCs through the use of an open-ended questionnaire administered to caregivers. This approach may underestimate the presence of PCCs compared with detailed report forms, such as those used in a Russian pediatric study^9^ and several adult-focused studies.^32,33^ However, the latter approach may overestimate symptom prevalence, especially when control group comparisons are not used to contextualize the findings. Also, we cannot exclude the possibility that our findings are associated with unmeasured and residual confounding, or that the small number of events for some outcomes limited our ability to detect some associations with SARS-CoV-2 test result status. Third, although approximately 20% of participants were lost to follow-up, this is lower than earlier reports and any differences between those who were and were not lost to follow-up were unlikely to be substantially associated with our findings or conclusions. We also could not match SARS-CoV-2–positive and SARS-CoV-2–negative children by study site, as some sites enrolled too few children; thus, country was used to perform matching. Additionally, we cannot exclude the possibility that biases may have influenced our findings. Knowledge of a prior infection could account for the small differences detected between SARS-CoV-2–positive and SARS-CoV-2–negative children.^6,27^ In addition, the symptoms that led to SARS-CoV-2 testing, our matching of SARS-CoV-2–positive and SARS-CoV-2–negative participants, and our losses to follow-up may have influenced our findings.

We did not perform antibody testing at 90 days to confirm the absence of SARS-CoV-2 infections in control participants. However, control group contamination during the 3-month follow-up would have been minimal, as seroprevalence studies in the United States and Canada indicate that, through the middle of 2021, few children had serologic evidence of prior SARS-CoV-2 infection.^10,34,35^ Furthermore, control group contamination would have biased the results toward a smaller difference in PCCs between groups. Finally, as participants were enrolled prior to January 20, 2021, our findings cannot be extrapolated to emerging SARS-CoV-2 variants of concern and to children who do not seek ED care, as prior suspicion of infection that was sufficient to bring the child to the ED could limit the generalizability of our findings to the latter population.

## Conclusions

This cohort study found that, although 10% of children hospitalized with acute SARS-CoV-2 infections and 5% of those discharged from the ED reported PCCs at 90 days, these rates were only slightly higher than the rates among SARS-CoV-2–negative controls. Risk factors for reporting PCCs included the number of acute symptoms, length of hospitalization, and older age. These findings can inform public health policy decisions regarding COVID-19 mitigation strategies for children and screening approaches for PCCs among those with severe infections.

## Notes

Supplement 1. **eAppendix.** Severe Outcomes **eTable 1.** Demographic and Clinical Characteristics of SARS-CoV-2-Positive Children by Follow-up Status at 90 Days **eTable 2.** Baseline Demographics of Participants and Presence of Self-Reported Persistent, New or Recurring Health Problems 90 Days Following ED Visit for 1884 SARS-CoV-2-Positive Children Enrolled Before January 20, 2021 **eTable 3.** Characteristics of Self-Reported Persistent, New or Recurring Health Problems 90 Days Following the Index ED Visit for SARS-CoV-2-Positive Participants **eTable 4.** Demographic and Clinical Characteristics (at Time of ED Presentation) of Frequency Matched SARS-CoV-2-Positive and SARS-CoV-2-Negative Children With Complete Follow-up, by Acute Hospitalization Status **eTable 5.** Comparison of Each Reported Type of Persistent, New or Recurring Health Problem Reported Among Frequency Matched SARS-CoV-2-Positive and Negative Participants With Complete 90-Day Follow-up **eTable 6.** Results of Multiple Logistic Regression Models Examining the Association of SARS-CoV-2 Positivity With Categories of Reported 90-Day Persistent, New or Recurring Health Problems **eFigure 1.** Types of 90-Day Persistent, New or Recurring Health Problem According to Number of Health Problems Reported Among the 110 SARS-CoV-2-Positive Children Reporting Any Persistent New or Recurring Health Problem **eFigure 2.** Follow-up of Frequency Matched SARS-CoV-2-Positive and Negative Children Who Were Not Hospitalized During the Acute Phase (Day 0-14) of Illness **eFigure 3.** Follow-up of Frequency Matched SARS-CoV-2-Positive and Negative Children Who Were Hospitalized During the Acute Phase (Day 0-14) of Illness

Supplement 2.
Nonauthor Collaborators

## References

[1] World Health Organization. Expanding our understanding of post COVID-19 condition: report of a WHO webinar—9 February 2021. Accessed May 24, 2022. https://www.who.int/publications/i/item/9789240025035

[2] Centers for Disease Control and Prevention. Post-COVID conditions: information for healthcare providers. Accessed August 10, 2021. https://www.cdc.gov/coronavirus/2019-ncov/hcp/clinical-care/post-covid-conditions.html?CDC_AA_refVal=https%3A%2F%2Fwww.cdc.gov%2Fcoronavirus%2F2019-ncov%2Fhcp%2Fclinical-care%2Flate-sequelae.html

[3] Lopez-Leon S, Wegman-Ostrosky T, Perelman C, . More than 50 long-term effects of COVID-19: a systematic review and meta-analysis. Sci Rep. 2021;11(1):16144. doi:10.1038/s41598-021-95565-8 34373540 PMC8352980

[4] Nasserie T, Hittle M, Goodman SN. Assessment of the frequency and variety of persistent symptoms among patients with COVID-19: a systematic review. JAMA Netw Open. 2021;4(5):e2111417. doi:10.1001/jamanetworkopen.2021.11417 34037731 PMC8155823

[5] Thomson H. Children with long COVID. New Sci. 2021;249(3323):10-11. doi:10.1016/S0262-4079(21)00303-1 33686318 PMC7927578

[6] Matta J, Wiernik E, Robineau O, ; Santé, Pratiques, Relations et Inégalités Sociales en Population Générale Pendant la Crise COVID-19–Sérologie (SAPRIS-SERO) Study Group. Association of self-reported COVID-19 infection and SARS-CoV-2 serology test results with persistent physical symptoms among French adults during the COVID-19 pandemic. JAMA Intern Med. 2022;182(1):19-25. doi:10.1001/jamainternmed.2021.6454 34747982 PMC8576624

[7] Buonsenso D, Munblit D, De Rose C, . Preliminary evidence on long COVID in children. Acta Paediatr. 2021;110(7):2208-2211. doi:10.1111/apa.15870 33835507 PMC8251440

[8] Smane L, Stars I, Pucuka Z, Roge I, Pavare J. Persistent clinical features in paediatric patients after SARS-CoV-2 virological recovery: a retrospective population-based cohort study from a single centre in Latvia. BMJ Paediatr Open. 2020;4(1):e000905. doi:10.1136/bmjpo-2020-000905 34192185 PMC7778738

[9] Osmanov IM, Spiridonova E, Bobkova P, ; Sechenov StopCOVID Research Team. Risk factors for long COVID in previously hospitalised children using the ISARIC global follow-up protocol: a prospective cohort study. Eur Respir J. 2021;59(2):2101341. doi:10.1183/13993003.01341-2021 34210789 PMC8576804

[10] Molteni E, Sudre CH, Canas LS, . Illness duration and symptom profile in symptomatic UK school-aged children tested for SARS-CoV-2. Lancet Child Adolesc Health. 2021;5(10):708-718. doi:10.1016/S2352-4642(21)00198-X 34358472 PMC8443448

[11] Chevinsky JR, Tao G, Lavery AM, . Late conditions diagnosed 1-4 months following an initial coronavirus disease 2019 (COVID-19) encounter: a matched-cohort study using inpatient and outpatient administrative data—United States, 1 March-30 June 2020. Clin Infect Dis. 2021;73(suppl 1):S5-S16. doi:10.1093/cid/ciab338 33909072 PMC8135331

[12] Blankenburg J, Wekenborg MK, Reichert J, . Comparison of mental health outcomes in seropositive and seronegative adolescents during the COVID19 pandemic. Sci Rep. 2022;12(1):2246. doi:10.1038/s41598-022-06166-y 35145161 PMC8831534

[13] Fazel M, Puntis S, White SR, . Willingness of children and adolescents to have a COVID-19 vaccination: results of a large whole schools survey in England. EClinicalMedicine. 2021;40:101144. doi:10.1016/j.eclinm.2021.101144 34608453 PMC8482530

[14] Klassen TP, Acworth J, Bialy L, ; PERN. Pediatric emergency research networks: a global initiative in pediatric emergency medicine. Pediatr Emerg Care. 2010;26(8):541-543. doi:10.1097/PEC.0b013e3181e5bec1 20657343

[15] Funk AL, Florin TA, Dalziel SR, . Prospective cohort study of children with suspected SARS-CoV-2 infection presenting to paediatric emergency departments: a Paediatric Emergency Research Networks (PERN) Study Protocol. BMJ Open. 2021;11(1):e042121. doi:10.1136/bmjopen-2020-042121 33452195 PMC7813043

[16] Vandenbroucke JP, von Elm E, Altman DG, ; STROBE initiative. Strengthening the Reporting of Observational Studies in Epidemiology (STROBE): explanation and elaboration. Ann Intern Med. 2007;147(8):W163-94. doi:10.7326/0003-4819-147-8-200710160-00010-w1 17938389

[17] Funk AL, Florin TA, Kuppermann N, ; Pediatric Emergency Research Network–COVID-19 Study Team. Outcomes of SARS-CoV-2–positive youths tested in emergency departments: the Global PERN-COVID-19 Study. JAMA Netw Open. 2022;5(1):e2142322. doi:10.1001/jamanetworkopen.2021.42322 35015063 PMC8753506

[18] Hoenig JM, Heisey DM. The abuse of power. Am Stat. 2001;55(1):19-24. doi:10.1198/000313001300339897

[19] Schnadower D, Kuppermann N, Macias CG, ; American Academy of Pediatrics Pediatric Emergency Medicine Collaborative Research Committee. Febrile infants with urinary tract infections at very low risk for adverse events and bacteremia. Pediatrics. 2010;126(6):1074-1083. doi:10.1542/peds.2010-0479 21098155

[20] Benjamini Y, Hochberg Y. Controlling the false discovery rate: a practical and powerful approach to multiple testing. J R Stat Soc B. 1995;57(1):289-300. doi:10.1111/j.2517-6161.1995.tb02031.x

[21] Denina M, Pruccoli G, Scolfaro C, . Sequelae of COVID-19 in hospitalized children: a 4-months follow-up. Pediatr Infect Dis J. 2020;39(12):e458-e459. doi:10.1097/INF.0000000000002937 33003103

[22] Zhang C, Huang L, Tang X, Zhang Y, Zhou X. Pulmonary sequelae of pediatric patients after discharge for COVID-19: an observational study. Pediatr Pulmonol. 2021;56(5):1266-1269. doi:10.1002/ppul.25239 33559979 PMC8012994

[23] Buonsenso D, Munblit D, De Rose C, . Preliminary evidence on long COVID in children. *medRxiv*. Preprint posted online January 26, 2021. doi:10.1101/2021.01.23.21250375 PMC8251440 33835507

[24] Stephenson T, Pinto Pereira S, Shafran R, ; CLoCk Consortium. Long COVID—the physical and mental health of children and non-hospitalised young people 3 months after SARS-CoV-2 infection; a national matched cohort study (The CLoCk) Study. Research Square. Posted August 10, 2021. Accessed October 13, 2021. https://assets.researchsquare.com/files/rs-798316/v1/82480913-3b6d-47fc-9d50-096244918954.pdf?c=1632402660 doi:10.21203/rs.3.rs-798316/v1

[25] Say D, Crawford N, McNab S, Wurzel D, Steer A, Tosif S. Post-acute COVID-19 outcomes in children with mild and asymptomatic disease. Lancet Child Adolesc Health. 2021;5(6):e22-e23. doi:10.1016/S2352-4642(21)00124-3 33891880 PMC8057863

[26] Miller F, Nguyen V, Navaratnam AM, . Prevalence of persistent symptoms in children during the COVID-19 pandemic: evidence from a household cohort study in England and Wales. medRxiv. Preprint posted online June 2, 2021. doi:10.1101/2021.05.28.21257602 PMC9645448 36375098

[27] Radtke T, Ulyte A, Puhan MA, Kriemler S. Long-term symptoms after SARS-CoV-2 infection in children and adolescents. JAMA. 2021;326(9):869-871. doi:10.1001/jama.2021.11880 34264266 PMC8283661

[28] Sudre CH, Murray B, Varsavsky T, . Attributes and predictors of long COVID. Nat Med. 2021;27(4):626-631. doi:10.1038/s41591-021-01292-y 33692530 PMC7611399

[29] Carfì A, Bernabei R, Landi F; Gemelli Against COVID-19 Post-Acute Care Study Group. Persistent symptoms in patients after acute COVID-19. JAMA. 2020;324(6):603-605. doi:10.1001/jama.2020.12603 32644129 PMC7349096

[30] Townsend L, Dyer AH, Jones K, . Persistent fatigue following SARS-CoV-2 infection is common and independent of severity of initial infection. PLoS One. 2020;15(11):e0240784. doi:10.1371/journal.pone.0240784 33166287 PMC7652254

[31] Dugdale CM, Anahtar MN, Chiosi JJ, . Clinical, laboratory, and radiologic characteristics of patients with initial false-negative severe acute respiratory syndrome coronavirus 2 nucleic acid amplification test results. Open Forum Infect Dis. 2020;8(1):ofaa559. doi:10.1093/ofid/ofaa559 34164560 PMC7717411

[32] Havervall S, Rosell A, Phillipson M, . Symptoms and functional impairment assessed 8 months after mild COVID-19 among health care workers. JAMA. 2021;325(19):2015-2016. doi:10.1001/jama.2021.5612 33825846 PMC8027932

[33] Trinkmann F, Müller M, Reif A, ; Lung Network Rhine-Neckar-Region. Residual symptoms and lower lung function in patients recovering from SARS-CoV-2 infection. Eur Respir J. 2021;57(2):2003002. doi:10.1183/13993003.03002-2020 33479105 PMC7821834

[34] Statistics Canada. Few Canadians had antibodies against SARS-CoV-2 in early 2021. Accessed August 10, 2021. https://www150.statcan.gc.ca/n1/en/daily-quotidien/210706/dq210706a-eng.pdf?st=bYnuCaHs

[35] Couture A, Lyons C, Mehrotra ML, . Methods for estimation of SARS-CoV-2 seroprevalence and reported COVID-19 cases in U.S. children, August 2020–May 2021. *medRxiv*. Preprint posted online September 29, 2021. doi:10.1101/2021.09.26.21263756

