# Error in Byline

**DOI:** 10.1001/jamanetworkopen.2022.31131

**Published:** 2022-08-18

In the Original Investigation titled “Post–COVID-19 Conditions Among Children 90 Days After SARS-CoV-2 Infection,”^1^ published July 22, 2022, the 51st author’s surname was misspelled; “Muhammad Wassem” should be changed to “Muhammad Waseem.” This article has been corrected.^1^
