# Supplementary material for: Post–COVID-19 Conditions Among Children 90 Days After SARS-CoV-2 Infection
Source: JAMA Netw Open. 2022 Jul 22;5(7):e2223253. doi: 10.1001/jamanetworkopen.2022.23253 (PMC9308058; doi:10.1001/jamanetworkopen.2022.23253)
Supplement: Supplement 1. — eAppendix. Severe Outcomes eTable 1. Demographic and Clinical Characteristics of SARS-CoV-2-Positive Children by Follow-up Status at 90 Days eTable 2. Baseline Demographics of Participants and Presence of Self-Reported Persistent, New or Recurring Health Problems 90 Days Following ED Visit for 1884 SARS-CoV-2-Positive Children Enrolled Before January 20, 2021 eTable 3. Characteristics of Self-Reported Persistent, New or Recurring Health Problems 90 Days Following the Index ED Visit for SARS-CoV-2-Positive Participants eTable 4. Demographic and Clinical Characteristics (at Time of ED Presentation) of Frequency Matched SARS-CoV-2-Positive and SARS-CoV-2-Negative Children With Complete Follow-up, by Acute Hospitalization Status eTable 5. Comparison of Each Reported Type of Persistent, New or Recurring Health Problem Reported Among Frequency Matched SARS-CoV-2-Positive and Negative Participants With Complete 90-Day Follow-up eTable 6. Results of Multiple Logistic Regression Models Examining the Association of SARS-CoV-2 Positivity With Categories of Reported 90-Day Persistent, New or Recurring Health Problems eFigure 1. Types of 90-Day Persistent, New or Recurring Health Problem According to Number of Health Problems Reported Among the 110 SARS-CoV-2-Positive Children Reporting Any Persistent New or Recurring Health Problem eFigure 2. Follow-up of Frequency Matched SARS-CoV-2-Positive and Negative Children Who Were Not Hospitalized During the Acute Phase (Day 0-14) of Illness eFigure 3. Follow-up of Frequency Matched SARS-CoV-2-Positive and Negative Children Who Were Hospitalized During the Acute Phase (Day 0-14) of Illness [file jamanetwopen-e2223253-s001.pdf]

Supplementary Online Content

Funk AL, Kuppermann N, Florin TA, et al; Pediatric Emergency Research Network–COVID-19 Study Team. Post–COVID-19 conditions among children 90 days after SARS-CoV-2 infection. *JAMA Netw Open.* 2022;5(7):e2223253. doi:10.1001/jamanetworkopen.2022.23253

eAppendix. Severe Outcomes
eTable 1. Demographic and Clinical Characteristics of SARS-CoV-2-Positive Children by Follow-up Status at 90 Days
eTable 2. Baseline Demographics of Participants and Presence of Self-Reported Persistent, New or Recurring Health Problems 90 Days Following ED Visit for 1884 SARS-CoV-2-Positive Children Enrolled Before January 20, 2021
eTable 3. Characteristics of Self-Reported Persistent, New or Recurring Health Problems 90 Days Following the Index ED Visit for SARS-CoV-2-Positive Participants
eTable 4. Demographic and Clinical Characteristics (at Time of ED Presentation) of Frequency Matched SARS-CoV-2-Positive and SARS-CoV-2-Negative Children With Complete Follow-up, by Acute Hospitalization Status
eTable 5. Comparison of Each Reported Type of Persistent, New or Recurring Health Problem Reported Among Frequency Matched SARS-CoV-2-Positive and Negative Participants With Complete 90-Day Follow-up
eTable 6. Results of Multiple Logistic Regression Models Examining the Association of SARS-CoV-2 Positivity With Categories of Reported 90-Day Persistent, New or Recurring Health Problems
eFigure 1. Types of 90-Day Persistent, New or Recurring Health Problem According to Number of Health Problems Reported Among the 110 SARS-CoV-2-Positive Children Reporting Any Persistent New or Recurring Health Problem
eFigure 2. Follow-up of Frequency Matched SARS-CoV-2-Positive and Negative Children Who Were Not Hospitalized During the Acute Phase (Day 0-14) of Illness
eFigure 3. Follow-up of Frequency Matched SARS-CoV-2-Positive and Negative Children Who Were Hospitalized During the Acute Phase (Day 0-14) of Illness

This supplementary material has been provided by the authors to give readers additional information about their work.

© 2022 Funk AL et al. *JAMA Network Open.*

eAppendix. Severe Outcomes

A priori, a severe outcome was defined by the occurrence of any of the following complications: cardiac or cardiovascular (cardiac arrest, cardiac ischemia, congestive heart failure, endocarditis, myocarditis, pericarditis, stroke), infectious (disseminated intravascular coagulation, mastoiditis, sepsis with bacteremia, septic shock, toxic shock syndrome), neurologic (encephalitis, meningitis), respiratory (acute respiratory distress syndrome, empyema, necrotizing or cryptogenic organizing pneumonia, pleural effusion or pneumothorax or pneumomediastinum requiring drainage, respiratory failure), and death. In addition, in the absence of one of the above events, performance of any of the following interventions also was deemed to represent a severe outcome: chest drainage, extracorporeal membrane oxygenation, inotropic support, positive pressure ventilation (invasive or noninvasive), and renal replacement therapy. The diagnosis of multi-system inflammatory syndrome in children (MIS-C) and Kawasaki disease were reported as assigned by the clinical care teams and were considered severe if accompanied by one of the above diagnoses or interventions.

© 2022 Funk AL et al. *JAMA Network Open.*

eTable 1. Demographic and Clinical Characteristics of SARS-CoV-2-Positive Children by Follow-up Status at 90 Days. All data are n (%) unless otherwise specified.

	Complete follow-up<br>N= 1884	Lost to follow-up<br>N= 289	Follow-up not attempted<br>N= 192
**Region**			
United States	1204 (63.9)	234 (81.0)	117 (60.9)
Costa Rica	331 (17.6)	21 (7.3)	5 (2.6)
Canada	172 (9.1)	19 (6.6)	38 (19.8)
Spain	133 (7.1)	0	1 (0.5)
Other[a]	44 (2.3)	15 (5.2)	31 (16.1)
**Sex, Male**	994 (52.8)	149 (51.6)	92 (47.9)
**Age**			
<1.0 yrs	490 (26.0)	79 (27.3)	57 (29.7)
1.0 - <2.0 yrs	232 (12.3)	32 (11.1)	22 (11.5)
2.0 - <5.0 yrs	295 (15.7)	51 (17.7)	31 (16.2)
5.0 - <10.0 yrs	364 (19.3)	49 (17.0)	34 (17.7)
10.0 - <14.0 yrs	240 (12.7)	41 (14.2)	25 (13.0)
14.0 - <18.0 yrs	263 (14.0)	37 (12.8)	23 (12.0)
**Chronic underlying condition, Yes**	270 (14.3)	35 (12.1)	17 (8.9)
**History of asthma, Yes**	260 (13.8)	42 (14.5)	18 (9.4)
**Number of symptoms at ED presentation**			
None	113 (6.0)	16 (5.5)	12 (6.3)
1-3	754 (40.0)	86 (29.8)	56 (29.2)
4-6	626 (33.2)	100 (34.6)	67 (34.9)
7 or more	391 (20.8)	87 (30.1)	56 (29.2)
**Types of symptoms[b]**			
Any systemic symptom	1478 (78.5)	239 (82.7)	163 (84.9)
Any respiratory symptom	1328 (70.5)	208 (72.0)	137 (71.4)
Any gastrointestinal symptom	749 (39.8)	147 (50.9)	89 (46.4)
Any neurological symptom	538 (28.6)	100 (34.6)	55 (28.7)
Any other symptom	526 (27.9)	136 (47.1)	84 (43.8)

ED, Emergency Department.

[a] Includes Argentina (n=13), Italy (n=1), Paraguay (n=28), and Singapore (n=2).

[b] Information was missing for a maximum of 3 children for each specific symptom type, unless otherwise noted

© 2022 Funk AL et al. *JAMA Network Open.*

eTable 2. Baseline Demographics of Participants and Presence of Self-Reported Persistent, New or Recurring Health Problems 90 Days Following ED Visit for 1884 SARS-CoV-2-Positive Children Enrolled Before January 20, 2021. Data are n/N (%).

	All children	Acute (day 0-14) SARS-CoV-2 outcome		P Value
		**Not hospitalized**	**Hospitalized**	
**All children**	**110/1884 (5.8)**	**66/1437 (4.6)**	**44/447 (9.8)**	**<0.001**
**Region**				
United States	**79/1204** (6.6)	45/927 (4.9)	34/277 (12.3)	<0.001
Costa Rica	**11/331** (3.3)	3/232 (1.3)	8/99 (8.1)	0.004
Canada	**17/172** (9.9)	15/158 (9.5)	2/14 (14.3)	0.63
Spain	**3/133** (2.3)	3/101 (3.0)	0/32 (0)	>0.99
Other[a]	**0/44** (0)	0/19 (0)	0/25 (0)	-
**Sex**				
Male	**53/994** (5.3)	33/748 (4.4)	20/246 (8.1)	0.03
Female	**57/890** (6.4)	33/689 (4.8)	24/201 (11.9)	<0.001
**Age**				
<1.0 yrs	**19/490** (3.9)	12/373 (3.2)	7/117 (6.0)	0.18
1.0 - <2.0 yrs	**7/232** (3.0)	5/191 (2.6)	2/41 (4.9)	0.61
2.0 - <5.0 yrs	**11/295** (3.7)	6/221 (2.7)	5/74 (6.8)	0.75
5.0 - <10.0 yrs	**19/364** (5.2)	16/289 (5.5)	3/75 (4.0)	0.78
10.0 - <14.0 yrs	**20/240** (8.3)	11/181 (6.1)	16/182 (8.8)	0.42
14.0 - <18.0 yrs	**34/263** (12.9)	9/59 (15.3)	18/81 (22.2)	0.39
**Chronic condition**				
Yes	**23/270** (8.5)	12/155 (7.7)	11/115 (9.6)	0.66
No	**87/1613** (5.4)	54/1281 (4.2)	33/332 (9.9)	<0.001
**Number of symptoms at ED presentation**				
None	**4/113** (3.5)	1/80 (1.3)	3/33 (9.1)	0.07
1-3	**17/754** (2.3)	9/585 (1.5)	8/169 (4.7)	0.03
4-6	**34/626** (5.4)	23/478 (4.8)	11/148 (7.4)	0.22
7 or more	**55/391** (14.1)	33/294 (11.2)	22/97 (22.7)	0.007
**Severe acute illness**				
Yes	**13/70** (18.6)	-	13/70 (18.6)	-

© 2022 Funk AL et al. *JAMA Network Open.*

No	**97/1814** (5.4)	66/1437 (4.6)	31/377 (8.2)	0.01

ED, Emergency Department.

[a] Includes Argentina (n=13), Italy (n=1), Paraguay (n=28), and Singapore (n=2).

© 2022 Funk AL et al. *JAMA Network Open*.

eTable 3. Characteristics of Self-Reported Persistent, New or Recurring Health Problems 90 Days Following the Index ED Visit for SARS-CoV-2-Positive Participants. All data are n (%) unless otherwise specified.

	**All children N=1884**	**Acute (day 0-14) SARS-CoV-2 Outcome**	
		**Not hospitalized N=1437**	**Hospitalized N=447**
**Persistent, new or recurring health problem, n (%) [95%CI]**	**110 (5.8)** [4.8-7.0]	**66/1437 (4.6)** [3.6-5.8]	**44/447 (9.8)** [7.2-13.0]
**Number of persistent, new or recurring health problem**			
1	65 (3.5)	37 (2.6)	28 (6.3)
2	25 (1.3)	17 (1.2)	8 (1.8)
3+	20 (1.1)	12 (0.8)	8 (1.8)
**Types of persistent, new or recurring health problem**			
**Respiratory, n (%) [95%CI]**	**38 (2.0)** [1.4-2.8]	**27 (1.9)** [1.2-2.7]	**11 (2.5)** [1.2-4.4]
Cough	13 (0.7)	9 (0.6)	4 (0.9)
Difficulty breathing, short of breath	13 (0.7)	10 (0.7)	3 (0.7)
Wheeze or asthma exacerbation	8 (0.4)	7 (0.5)	1 (0.2)
Runny nose or congestion	6 (0.3)	4 (0.3)	2 (0.5)
Chest pain	3 (0.2)	3 (0.2)	0
Other respiratory symptoms or diagnoses	12 (0.6)	5 (0.4)	7 (1.6)
**Systemic, n (%) [95%CI]**	**33 (1.8)** [1.2-2.5]	**24 (1.7)** [1.1-2.5]	**9 (2.0)** [0.9-3.8]
Fatigue or weakness	21 (1.1)	14 (1.0)	7 (1.6)
Fever	9 *(0.5)*	7 (0.5)	2 (0.5)
Anorexia, loss of appetite	7 (0.4)	7 (0.5)	0
Muscle, joint, or body pain	4 (0.2)	3 (0.2)	1 (0.2)
Mental 'fuzziness', loss of focus	4 *(0.2)*	3 *(0.2)*	1 *(0.2)*
Dizziness or lightheaded	2 (0.1)	2 (0.1)	0
**Psychological, n (%) [95%CI]**	**17 (0.9)** [0.5-1.4]	**9 (0.6)** [0.3-1.2]	**8 (1.8)** [0.8-3.5]
Anxiety	7 (0.4)	3 (0.2)	4 (0.9)
Depression	6 (0.3)	3 (0.2)	3 (0.7)
Other psychological symptoms or diagnoses	7 (0.4)	3 (0.1)	4 (0.9)

© 2022 Funk AL et al. *JAMA Network Open.*

**Neurologic, n (%) [95%CI]**	**17 (0.9)** [0.5-1.4]	**10 (0.7)** [0.3-1.3]	**7 (1.6)** [0.6-3.2]
Loss of smell or taste	9 (0.5)	7 (0.5)	2 (0.5)
Headache	7 (0.4)	3 (0.2)	4 (0.9)
Seizures	1 (0.1)	0	1 (0.2)
**Gastrointestinal, n (%) [95%CI]**	**12 (0.6)** [0.3-1.1]	**4 (0.3)** [0.1-0.7]	**8 (1.8)** [0.8-3.5]
**Cardiovascular, n (%) [95%CI]**	**12 (0.6)** [0.3-1.1]	**3 (0.2)** [0-0.6]	**9 (2.0)** [0.9-3.8]
**Skin condition or rash, n (%) [95%CI]**	**10 (0.5)** [0.3-1.0]	**9 (0.6)** [0.3-1.2]	**1 (0.2)** [0-1.2]
**Ophthalmologic and/or otolaryngologic, n (%) [95%CI]**	**4 (0.2)** [0.1-0.5]	**2 (0.1)** [0-0.5]	**2 (0.5)** [0.1-1.6]
**Other symptoms or diagnoses, n (%) [95%CI]**	**12 (0.6)** [0.3-1.1]	**6 (0.4)** [0.2-0.9]	**6 (1.3)** [0.5-2.9]

© 2022 Funk AL et al. *JAMA Network Open*.

eTable 4. Demographic and Clinical Characteristics (at Time of ED Presentation) of Frequency Matched SARS-CoV-2-Positive and SARS-CoV-2-Negative Children With Complete Follow-up, by Acute Hospitalization Status. Data are n/N (%).

	**Not hospitalized within 14 days of testing**		**Hospitalized within 14 days of testing**	
	**SARS-CoV-2 positive N=1295**	**SARS-CoV-2 negative N=1321**	**SARS-CoV-2 positive N=391**	**SARS-CoV-2 negative N=380**
**Sex**, Male	667 (51.5)	622 (47.1)	210 (53.7)	197 (51.8)
**Age**				
<1.0 yrs	338 (26.1)	258 (19.5)	99 (25.3)	84 (22.1)
1.0 - <2.0 yrs	173 (13.4)	265 (20.1)	34 (8.7)	41 (10.8)
2.0 - <5.0 yrs	198 (15.3)	312 (23.6)	60 (15.4)	73 (19.2)
5.0 - <10.0 yrs	260 (20.1)	246 (18.6)	69 (17.7)	70 (18.4)
10.0 - <14.0 yrs	164 (12.7)	133 (10.1)	49 (12.5)	62 (16.3)
14.0 - <18.0 yrs	162 (12.5)	107 (8.1)	80 (20.5)	50 (13.2)
**Chronic underlying condition**	131 (10.1)	215 (16.3)	104 (26.6)	103 (27.1)
**History of asthma**	170 (13.1)	193 (14.6)	65 (16.6)	84 (22.1)
**Number of symptoms at ED presentation**				
None	73 (5.6)	97 (7.3)	24 (6.1)	24 (6.3)
1-3	513 (39.6)	472 (35.7)	149 (38.1)	153 (40.3)
4-6	442 (34.1)	475 (36.0)	128 (32.7)	139 (36.6)
7 or more	267 (20.6)	277 (21.0)	90 (23.0)	64 (16.8)
**Types of symptoms**				
Any systemic symptom	1027 (79.3)	1004 (76.0)	309 (79.0)	270 (71.1)
Any respiratory symptom	961 (74.2)	813 (61.5)	222 (56.8)	174 (45.8)
Any gastrointestinal symptom	506 (39.1)	583 (44.1)	182 (46.6)	180 (47.4)
Any neurological symptom	372 (28.7)	277 (21.0)	115 (29.4)	88 (23.2)
Any other symptom	483 (37.3)	589 (44.6)	147 (37.6)	158 (41.6)

ED, Emergency Department.

© 2022 Funk AL et al. *JAMA Network Open.*

eTable 5. Comparison of Each Reported Type of Persistent, New or Recurring Health Problem Reported Among Frequency Matched SARS-CoV-2-Positive and Negative Participants With Complete 90-Day Follow-up. Data are n/N (%).

	**Not acutely hospitalized**			**Acutely hospitalized**		
	**SARS-CoV-2 Positive N=1295**	**SARS-CoV-2 Negative N=1321**	**P Value**	**SARS-CoV-2 Positive N=391**	**SARS-CoV-2 Negative N=380**	**P Value**
**Respiratory**	**20 (1.8)**	**24 (1.5)**	**0.59**	**10 (2.6)**	**7 (1.8)**	**0.63**
Cough	8 (0.6)	11 (0.8)	0.65	4 (1.0)	2 (0.5)	0.69
Difficulty breathing, short of breath	8 (0.6)	4 (0.3)	0.26	3 (0.8)	0	0.25
Wheeze or asthma exacerbation	4 (0.3)	7 (0.5)	0.55	1 (0.3)	1 (0.3)	>0.99
Runny nose or congestion	3 (0.2)	6 (0.5)	0.51	2 (0.5)	1 (0.3)	>0.99
Chest pain	3 (0.2)	1 (0.1)	0.37	0	1 (0.3)	0.49
Sneezing	0	1 (0.1)	>0.99	0	0	-
Sore throat	0	1 (0.1)	>0.99	0	0	-
Other respiratory symptoms & diagnoses	4 (0.3)	3 (0.2)	0.72	6 (1.5)	3 (0.8)	0.51
**Systemic**	**21 (1.6)**	**6 (0.5)**	**0.003**	**8 (2.1)**	**5 (1.3)**	**0.58**
Fatigue or weakness	12 (0.9)	0	<0.001	6 (1.5)	1 (0.3)	0.12
Fever	6 (0.5)	5 (0.4)	0.77	2 (0.5)	0	0.50
Anorexia, loss of appetite	7 (0.5)	1 (0.1)	0.04	0	0	-
Muscle, joint, or body pain	3 (0.2)	0	0.12	1 (0.3)	2 (0.5)	0.62
Mental ‘fuzziness’, loss of focus	3 (0.2)	0	0.12	1 (0.3)	0	>0.99
Dizziness or lightheaded	2 (0.2)	1 (0.1)	0.62	0	2 (0.5)	0.24
**Psychological**	**9 (0.7)**	**2 (0.2)**	**0.04**	**8 (2.1)**	**5 (1.3)**	**0.58**
Anxiety	3 (0.2)	1 (0.1)	0.37	4 (1.0)	1 (0.3)	0.37
Depression	3 (0.2)	0	0.12	3 (0.8)	2 (0.5)	>0.99
Other psychological symptoms, diagnoses	2 (0.2)	0	0.25	4 (1.0)	3 (0.8)	>0.99
**Neurologic**	**9 (0.7)**	**5 (0.4)**	**0.30**	**6 (1.5)**	**4 (1.1)**	**0.75**
Loss of smell or taste	6 (0.5)	0	0.02	2 (0.5)	0	0.50
Headache	3 (0.2)	2 (0.2)	0.68	3 (0.8)	3 (0.8)	>0.99
Seizures	0	3 (0.2)	0.25	1 (0.3)	1 (0.3)	>0.99
**Gastrointestinal**	**4 (0.3)**	**4 (0.3)**	**>0.99**	**8 (2.0)**	**5 (1.3)**	**0.58**
**Cardiovascular**	**3 (0.2)**	**0**	**0.12**	**6 (1.5)**	**0**	**0.03**

© 2022 Funk AL et al. *JAMA Network Open.*

**Skin condition or rash**	**5 (0.4)**	**1 (0.1)**	**0.12**	**1 (0.3)**	**0**	**>0.99**
**Ophthalmologic and/or otolaryngologic**	**2 (0.2)**	**0**	**0.25**	**2 (0.5)**	**0**	**0.50**
**Other symptoms or diagnoses**	**5 (0.4)**	**4 (0.3)**	**0.75**	**5 (1.3)**	**2 (0.5)**	**0.45**

© 2022 Funk AL et al. *JAMA Network Open.*

eTable 6. Results of Multiple Logistic Regression Models Examining the Association of SARS-CoV-2 Positivity With Categories of Reported 90-Day Persistent, New or Recurring Health Problems.*

	**Number of events**	**Adjusted odds ratio (SARS-CoV-2) (95% confidence interval)**	**p-value**	**Covariates included in Final Models in Addition to SARS-CoV-2 Test Result**
**Any reported persistent, new or recurring health problem**	147	1.63 (1.14, 2.35)	**0.008**	Country of enrolment, sex, age category, having a chronic condition other than asthma acute hospitalization, # acute symptoms, season of infection
**Respiratory**	61	0.88 (0.52, 1.48)	0.62	Country of enrolment, age category, # acute symptoms
**Systemic**	40	2.44 (1.19, 5.00)	**0.02**	Having a chronic condition other than asthma, sex, age category, acute hospitalization, # acute symptoms
**Neurologic**	24	1.29 (0.56, 2.99)	0.55	Age category
**Psychological**	22	1.96 (0.74, 5.18)	0.17	Sex, age category, acute hospitalization, # acute symptoms
**Gastrointestinal**	21	1.35 (0.57, 3.21)	0.50	None

* Only persistent, new or recurring health problem categories for which there were at least 20 events were examined in a multiple logistic regression model. Acute hospitalization was included as a three-category variable: not hospitalized, hospitalized < 48 hours, hospitalized for ≥ 48 hours.

© 2022 Funk AL et al. *JAMA Network Open.*

eFigure 1. Types of 90-Day Persistent, New or Recurring Health Problem According to Number of Health Problems Reported Among the 110 SARS-CoV-2-Positive Children Reporting Any Persistent New or Recurring Health Problem

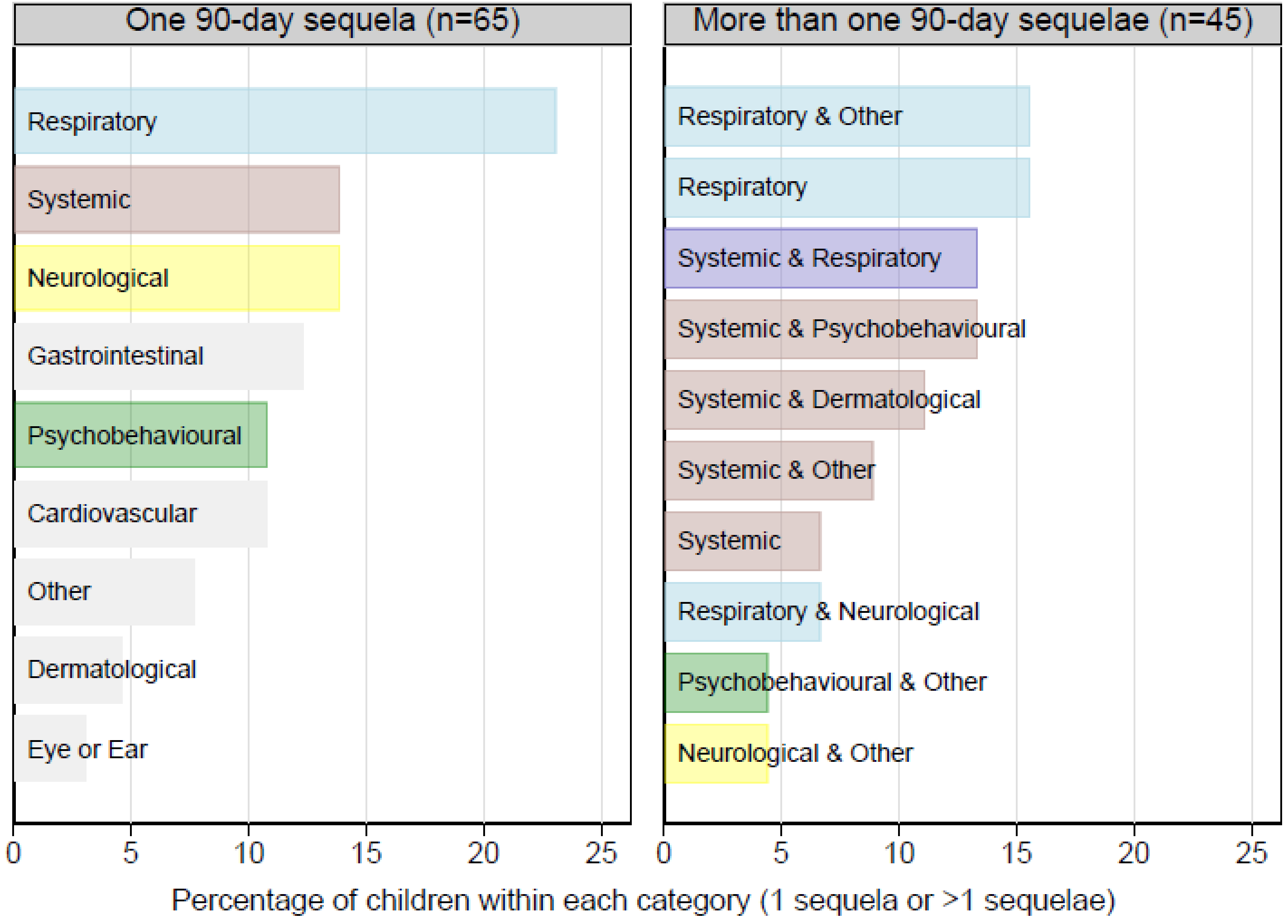

© 2022 Funk AL et al. *JAMA Network Open.*

eFigure 2. Follow-up of Frequency Matched SARS-CoV-2-Positive and Negative Children Who Were Not Hospitalized During the Acute Phase (Day 0-14) of Illness

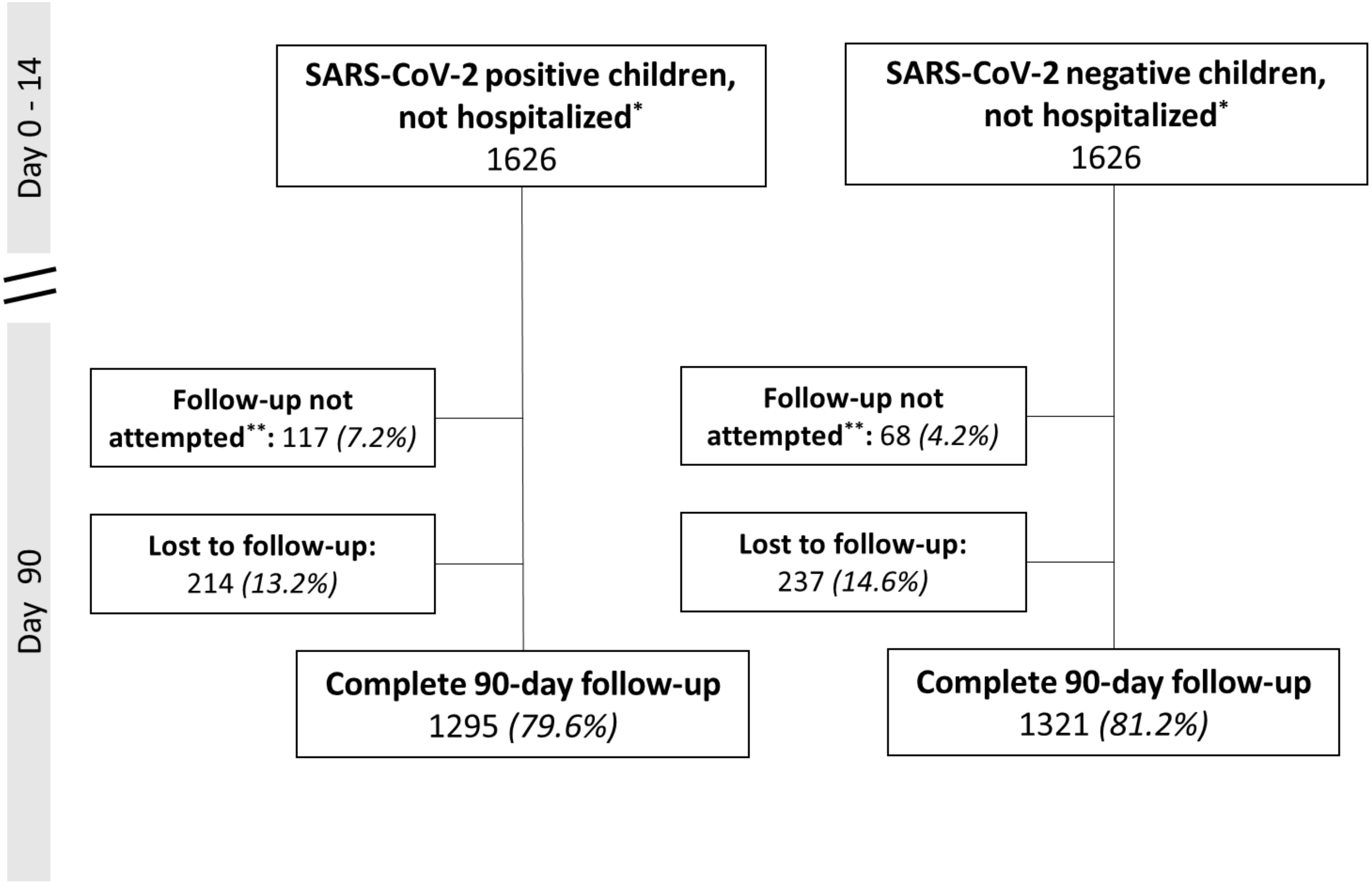

* Within 14 days of testing ** In a few sites, consistent 90-day follow-up was not feasible amid human resource constraints during the COVID-19 pandemic

© 2022 Funk AL et al. *JAMA Network Open.*

eFigure 3. Follow-up of Frequency Matched SARS-CoV-2-Positive and Negative Children Who Were Hospitalized During the Acute Phase (Day 0-14) of Illness

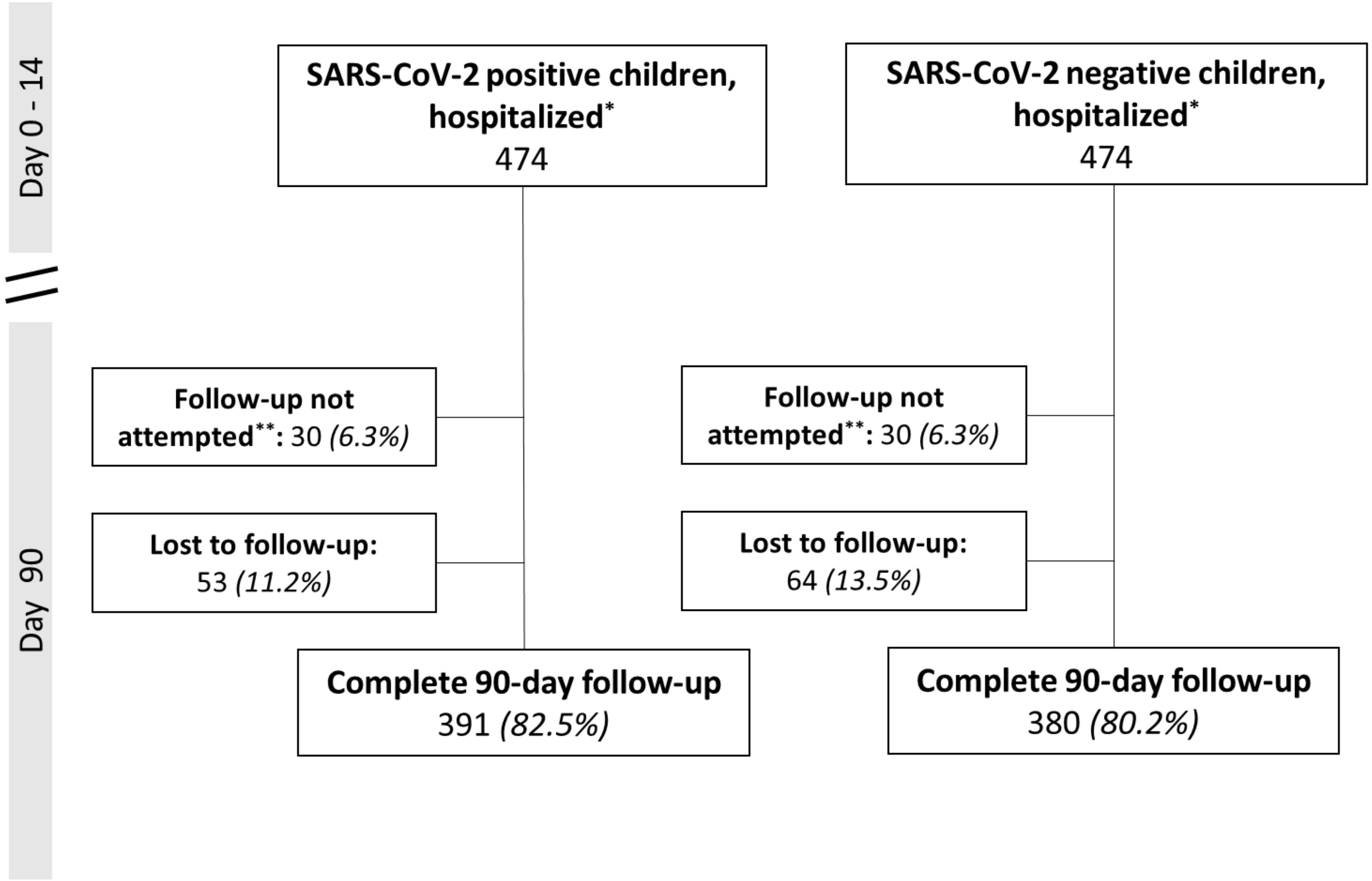

* Within 14 days of testing ** In a few sites, consistent 90-day follow-up was not feasible amid human resource constraints during the COVID-19 pandemic

© 2022 Funk AL et al. *JAMA Network Open.*
